# Supplementary material for: A plant RNA virus inhibits NPR1 sumoylation and subverts NPR1-mediated plant immunity
Source: Nat Commun. 2023 Jun 16;14:3580. doi: 10.1038/s41467-023-39254-2 (PMC10275998; doi:10.1038/s41467-023-39254-2)
Supplement: Supplementary file 1 — Supplementary inforation [file 41467_2023_39254_MOESM1_ESM.pdf]

A plant RNA virus inhibits NPR1 sumoylation and subverts NPR1-mediated plant immunity

Jiahui Liu, Xiaoyun Wu, Yue Fang, Ye Liu, Bello E. Oreofe, Yong Li, Ruyi Xiong, Yinzi Li, Zheng Qing Fu, Aiming Wang, Xiaofei Cheng*

Supplementary Table 1 Primers used in the present study.

Primer name	Sequence (5′-3′)	Usage
207-NPR1-F	GTACAAAAAAGCAGGCTTCATGGACACCACCATTGATGGATTC	Cloning
207-NPR1-R	CAAGAAAGCTGGGTCCCGACGCCGACGACGATGAGAGAGTTTAC	Cloning
207-GUS-F	GTACAAAAAAGCAGGCTTCATGTTACGTCCTGTAGAAACCCCAAC	Cloning
207-GUS-R	CAAGAAAGCTGGGTCCCGACGTTGTTTGCCTCCCTGCTGCGG	Cloning
207-SCMV NIb-F	GTACAAAAAAGCAGGCTTCATGTGCAAAATCACTGAAACTTG	Cloning
207-SCMV NIb-R	CAAGAAAGCTGGGTCCCGACGCTGGTGGAAAACTTCTTCGTTG	Cloning
207-PRSV NIb-F	GTACAAAAAAGCAGGCTTCATGAGTGGAAGTCGATGGCTTTTTG	Cloning
207-PRSV NIb-R	CAAGAAAGCTGGGTCCCGACGCCTGGTGAAACACATGTGTGTC	Cloning
207-PVMV NIb-F	GTACAAAAAAGCAGGCTTCATGGGAGAAGAAAAATGGTTG	Cloning
207-PVMV NIb-R	CAAGAAAGCTGGGTCCCGACGCTTGGTGCAGAACAAAGTCATC	Cloning
SMVNIb-dorF	GTACAAAAAAGCAGGCTTCATGGGGAAAAAGGAAAGATG	Cloning
SMVNIb-dorR	CAAGAAAGCTGGGTCCCGACGTTGCAAAGAAACTGATTCACAAC	Cloning
BCMVNIb-dorF	GTACAAAAAAGCAGGCTTCATGAGCAAGCAACAGTGGGTCTTG	Cloning
BCMVNIb-dorR	CAAGAAAGCTGGGTCCCGACGTTGTAGGTGCACAGATTCTCCGC	Cloning
PVYNIb-dorF	GTACAAAAAAGCAGGCTTCATGGCTAAGCACTCCGCGTGGATG	Cloning
PVYNIb-dorR	CAAGAAAGCTGGGTCCCGACGTTGATGGTATACTTCATAAGAG	Cloning
207-GmNPR1-F	GTACAAAAAAGCAGGCTTCATGGCTTATTCAGCCGAACCCTC	Cloning
207-GmNPR1-R	CAAGAAAGCTGGGTCCCGACGCCACTTTCCTAGCCTTGTAATGTAC	Cloning
207-SUMO3-F	GTACAAAAAAGCAGGCTTCATGTCTAACCCTCAAGATGAC	Cloning
207-GmSUMO1-F	GTACAAAAAAGCAGGCTTCATGTCGGGCGTTACCAACAACAAC	Cloning
207-GmSUMO1-R	CAAGAAAGCTGGGTCCCGACGGACAACAGAACCTCCTGTCTGG	Cloning
207-GmSUMO2-F	GTACAAAAAAGCAGGCTTCATGTCTGTATCAGGAGGCAGAG	Cloning
207-GmSUMO2-R	CAAGAAAGCTGGGTCCCGACGAGCATTTTGATGAAGATGATC	Cloning
207-GmSUMO4-F	GTACAAAAAAGCAGGCTTCATGGCTACCTCCAGAGGTCGAC	Cloning
207-GmSUMO4-R	CAAGAAAGCTGGGTCCCGACGACATCTCATGTCACCACCACC	Cloning
207-GmSUMO3-F	GTACAAAAAAGCAGGCTTCATGTCTGCATCAGGAGGCAGAG	Cloning
207-GmSUMO3-R	CAAGAAAGCTGGGTCCCGACGAAGAAACTTGTGTCCACCTCCG	Cloning
207-GmSUMO5-F	GTACAAAAAAGCAGGCTTCATGGCTACTAATGGTCCACTTAAG	Cloning
207-GmSUMO5-R	CAAGAAAGCTGGGTCCCGACGAAGTGCTGCAGCACCACCTCC	Cloning
207-GmSUMO6-F	GTACAAAAAAGCAGGCTTCATGGCTACCAATGGTCCACTTAAG	Cloning
207-GmSUMO6-R	CAAGAAAGCTGGGTCCCGACGAAGTGCCGCAGCACCACCTCC	Cloning
207-TGA3-F	GTACAAAAAAGCAGGCTTCATGGAGATGATGAGCTCTTC	Cloning
207-TGA3-R	CAAGAAAGCTGGGTCCCGACGAGTGTGTTCTCGTGGACGAGC	Cloning
207-TCP23-F	GTACAAAAAAGCAGGCTTCAATGGAGTCCCACAACAACAACC	Cloning
207-TCP23-R	CAAGAAAGCTGGGTCCCGACGAGGAGAACCATCTATAGTAGG	Cloning
207-TCP4-F	GTACAAAAAAGCAGGCTTCAATGTCTGACGACCAATTCCATC	Cloning
207-TCP4-R	CAAGAAAGCTGGGTCCCGACGATGGCGAGAAATAGAGGAAGCAG	Cloning

Supplementary Table 1 continued

Primer name	Sequence (5′-3′)	Usage
207-TCP3-F	GTACAAAAAAGCAGGCTTCAATGGCACCAGATAACGACCATTTC	Cloning
207-TCP3-R	CAAGAAAGCTGGGTCCCGACGATGGCGAGAATCGGATGAAGCAG	Cloning
207-FTM6-F	GTACAAAAAAGCAGGCTTCAATGGAGGGTAAGAGATCACAAG	Cloning
207-FTM6-R	CAAGAAAGCTGGGTCCCGACGTCTAATCTGTGGTCGCTTGAATG	Cloning
207-CRF6-F	GTACAAAAAAGCAGGCTTCAATGGAGAGACGAACGAGACGAG	Cloning
207-CRF6-R	CAAGAAAGCTGGGTCCCGACGATCGAAAGAGTGATGATGATGG	Cloning
207-BRM-F	GTACAAAAAAGCAGGCTTCAATGCAATCTGGAGGCAGTGGCG	Cloning
207-BRM-N-R	CAAGAAAGCTGGGTCCCGACGATTTTCTTTTGGCGCATTCATTTC	Cloning
207-FBH3-F	GTACAAAAAAGCAGGCTTCATGGAATCAGAATTCCAGCAAC	Cloning
207-FBH3-R	CAAGAAAGCTGGGTCCCGACGCGCACTAGAGCATCTACATC	Cloning
207-ATERF5-F	GTACAAAAAAGCAGGCTTCATGGCGACTCCTAACGAAGTATC	Cloning
207-ATERF5-R	CAAGAAAGCTGGGTCCCGACGAACAACGGTCAACTGGGAATAAC	Cloning
pGEX-F3	GGTCGACTCGAGCGGCCGC	Cloning
pGEX-R3	CGGGAATTCCGGGGATCCACG	Cloning
pGEX-NIb-R	GCGGCCGCTCGAGTCGACCCTGGTGATAAACACAAGCCTCAG	Cloning
pGEX-NIb-F	CGTGGATCCCCGGAATTCCCGATGACCCAGCAGAATCGGTGGATG	Cloning
NPR1F1_entF	AGGTAGTCATCAGATATATGGACACCACCATTGATGGATTC	Cloning
NPR1F1_entR	AGACTAAAGATGAGATCTAAAGACTTGACAATAATCTCTTTACATCT	Cloning
NPR1F2_entF	AGGTAGTCATCAGATATATGAATGTAGATATGGTTAGTCTTGA	Cloning
NPR1F2_entR	AGACTAAAGATGAGATCTAACTCGAGGCTAGTCACTATGAACTC	Cloning
NPR1F3_entF	AGGTAGTCATCAGATATATGCCTGACCGTCTCACTGGTACG	Cloning
NPR1F3_entR	AGACTAAAGATGAGATCTAACCGACGACGATGAGAGAGTTTACG	Cloning
NPR1-nLUC-F	GAGCTCGGTACCCGGGATCCATGGACACCACCATTGATGG	Cloning
NPR1-nLUC-R	CGAGATCTGGTCGACCCGACGACGATGAGAGAGTTTAC	Cloning
SUM3-cLUC-F	TCCCGGGGCGGTACCATGTCTAACCCTCAAGATGAC	Cloning
SUM3-cLUC-R	CTGCAGGTCGACTCTAGAAGCCCATTATGATCGAAAAGC	Cloning
CLUC-SUMO3dGG-R	CTGCAGGTCGACTCTAGTCATGCTGCACTCATCGCCCGGCACGCATC	Cloning
SMV-NIb-nLUC-F	GAGCTCGGTACCCGGGATCCATGGGGAAAAAGGAAAGATGGG	Cloning
SMV-NIb-nLUC-R	CGAGATCTGGTCGACTTGCAAAGAAACTGATTCACAAC	Cloning
cLUC-GmNPR1-F	TCCCGGGGCGGTACCATGGCTTATTCAGCCGAACC	Cloning
cLUC-GmNPR1-R	CTGCAGGTCGACTCTAGCACTTTCCTAGCCTTGTAATGTAC	Cloning
cLUC-TGA3-F	TCCCGGGGCGGTACCATGGAGATGATGAGCTCTTC	Cloning
cLUC-TGA3-R	CTGCAGGTCGACTCTAGAGTGTGTTCTCGTGGACGAGC	Cloning
NPR1promter-F	CTCAGAAGACCAAAGGGCTATGTTTACATATATTTATAGCTTAC	Cloning
NPR1promter-R	GAATCCATCAATGGTGGTGTCCATCAACAGGTTCCGATGAATTG	Cloning
NPR1-F	ATGGACACCACCATTGATGGATTC	Cloning
103-NPR1promter-R	ATAGCCCTTTGGTCTTCTGAG	Cloning

Supplementary Table 1 continued

Primer name	Sequence (5′-3′)	Usage
GmNPR1-pd-F	CATCCCTCAGAGATTCTGTTGTAC	Mutagenesis
GmNPR1-zt-R	CAACAGAATCTCTGAGGGATGATG	Mutagenesis
GmNPR1 sim3-F	GCAGCATCCGCACTTACGAAAGGGGCTTGTGC	Mutagenesis
GmNPR1 sim3-R	GTGCGGATGCTGCAATGGAAGGCTCTTTACGC	Mutagenesis
NPR1-zt-F	GACAGATTCGACTTCTTCCACATCG	Mutagenesis
NPR1-pd-R	GGAAGAAGTCGAATCTGTCAGGGAC	Mutagenesis
NPR1-c156a-pd-F	CGAGAATTGCGCACACGTGGCTTGCCGGCCG	Mutagenesis
NPR1-c156a-zt-R	CCACGTGTGCGCAATTCTCGTCTGCGC	Mutagenesis
NPR1-c82a-pd-F2	CTTTCCACCGGGCAGTTTTG	Mutagenesis
NPR1-c82a-zt-R3	AACTGCCCGGTGGAAAGAAACTTC	Mutagenesis
NPR1-c216a-pd-F	GGTAAAGCTGCAATGAAGCTATTGGATAGATG	Mutagenesis
NPR1-c216a-zt-R	GCTTCATTGCAGCTTTACCACATATATTAG	Mutagenesis
207-SUMO3dGG-R	CAAGAAAGCTGGGTCCCGACGTCATGCTGCACTCATCGCCCGGCACGCATC	Mutagenesis
NIbSM1-F	GCAGCAATAGCAGTATCAGATCTGCGAGGTTATG	Mutagenesis
221NIbSIM1-R	CTGATACTGCTATTGCTGCTGTCTTTTCAAACAGATCACAG	Mutagenesis
NIb409-F	CCCGGCTCGAGCCAGAGCGA	Mutagenesis
221NIb409-R	TCGCTCTGGCTCGAGCCGGGGAATCCAGATTCCCTCTCTTTTG	Mutagenesis
TuMVCPRTF	CAGGTTTGACAGACGAGCAA	qPCR
TuMVCPRTR	CCAGAGGTTCCAGCGTTTAC	qPCR
PR1-RTF	CTCATACACTCTGGTGGG	qPCR
PR1-RTR	TTGGCACATCCGAGTC	qPCR
ACTIN2-RTF	CCGGTATTGTGCTGGATTCT	qPCR
ACTIN2-RTR	TTCTCGATGGAAGAGCTGGT	qPCR
UBQ5-F	GTGGTGCTAAGAAGAGGAAGA	qPCR
UBQ5-R	TCAAGCTTCAACTCCTTCTTT	qPCR
GAPDH-F	TCTCGATCTCAATTTCGCAAAA	qPCR
GAPDH-R	CGAAACCGTTGATTCCGATTC	qPCR
npr1-0-LP-F	GAGCAGCGTCATCTTCAATTC	Genotyping
npr1-0-RP-R	TTGTCAGCGAGAAGCTCTTTC	Genotyping
Salk_LBb1.3	ATTTTGCCGATTTCGGAAC	Genotyping
npr1-6-RP	CTCTCAAAGGCCGACTATGTG	Genotyping
npr1-6-LP	ATTTGTTTGAAGCACACCTGC	Genotyping
SAIL_LBb3	TAGCATCTGAATTTCATAACCAATCTCGATACAC	Genotyping

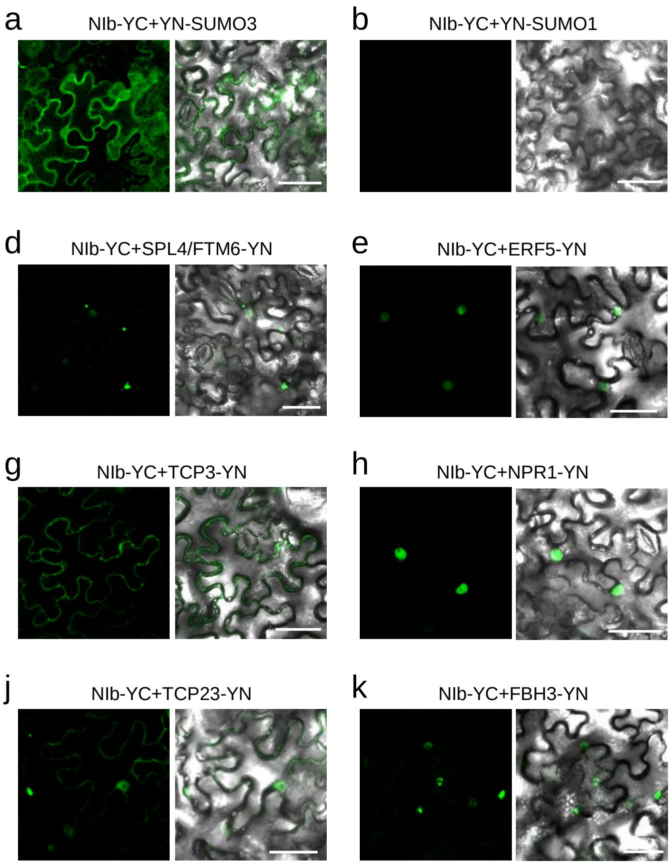

Supplementary Fig. 1 NIb interacts with SUMO3 substrates in the BiFC assay. a-k Confocal microscopic photographs of *N. benthamiana* epidermal cells expressing NIb-YC and C-terminal YN-tagged SUMO3 (**a**), SUMO1 (**b**), CRF6 (**c**), SPL4/FTM6 (**d**), ERF5 (**e**), TCP4 (**f**), TCP3 (**g**), NPR1 (**h**), the N-terminal part of BRM (BRM-N) (**i**), TCP23 (**j**), and FBH3 (**j**) at 2 dpi. Right panels showing the overlap of YFP and differential interference contrast channels. Scale bar, 50 μm. All experiments were independently repeated three times with similar results.

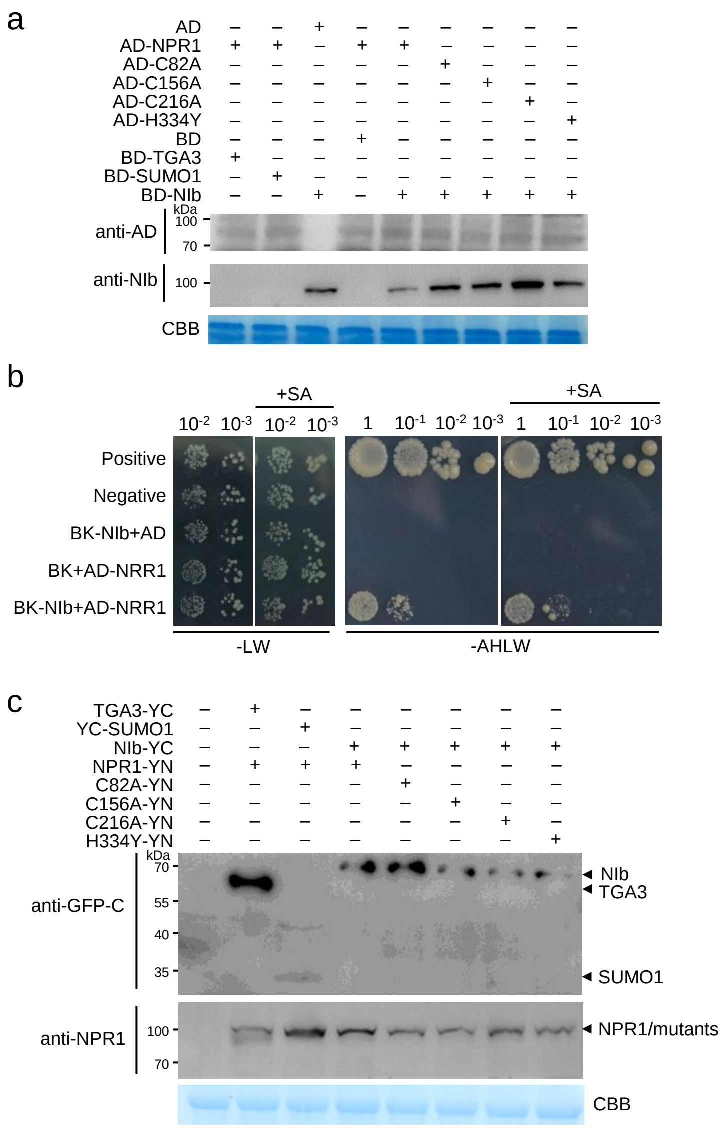

Supplementary Fig. 2 NIb interacts with NPR1. a Immunoblots for the expression of AD-tagged recombinant proteins and BD-NIb of the Y2H assay in Fig. 1a with anti-GAL4 AD (anti-AD) and anti-NIb antibodies, respectively. The experiment was independently repeated twice with similar results. **b** Yeast transformed with the indicated plasmids were serially diluted on selective medium supplied with or without 1 mM SA. Positive and negative controls are the same as in Fig. 1a. **c** Immunoblots for the expression of YC-tagged proteins and YN-tagged NPR1 or its mutants of the BiFC assay in Fig. 1b with anti-GFP-C and anti-NPR1 antibodies, respectively. The experiment was independently repeated three times with similar results.

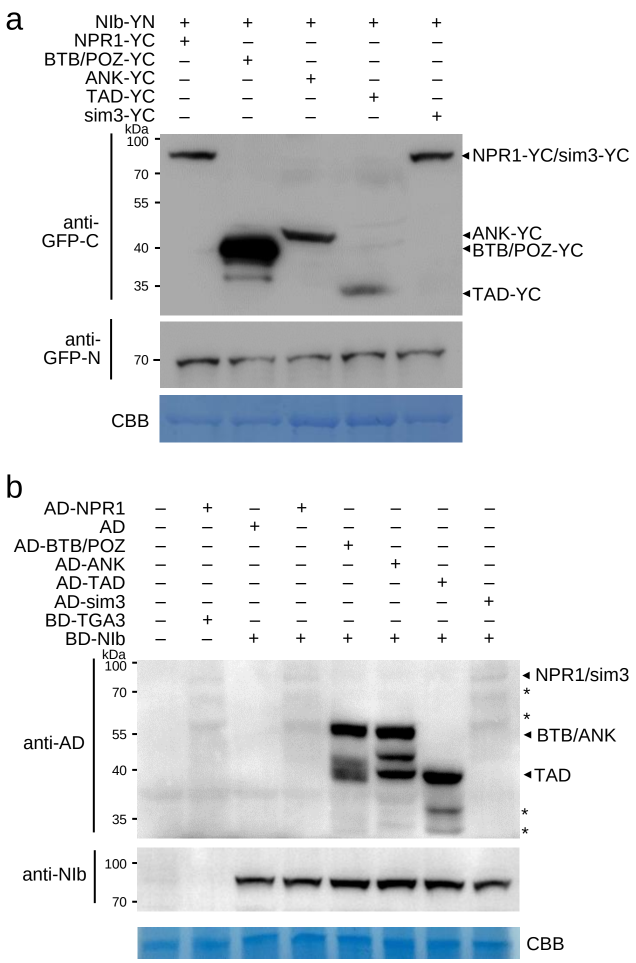

Supplementary Fig. 3 NIb targets SIM3 of NPR1. a Immunoblots for the expression of NIb-YN and YC-tagged NPR1 or mutants of the BiFC assay in Fig. 1f with anti-GFP-N and anti-GFP-C antibodies, respectively. **b** Immunoblots for the expression of BD-NIb and AD-tagged NPR1 or mutants of the Y2H assay in Fig. 1g with anti-NIb and anti-AD antibodies, respectively. All experiments were independently repeated three times with similar results.

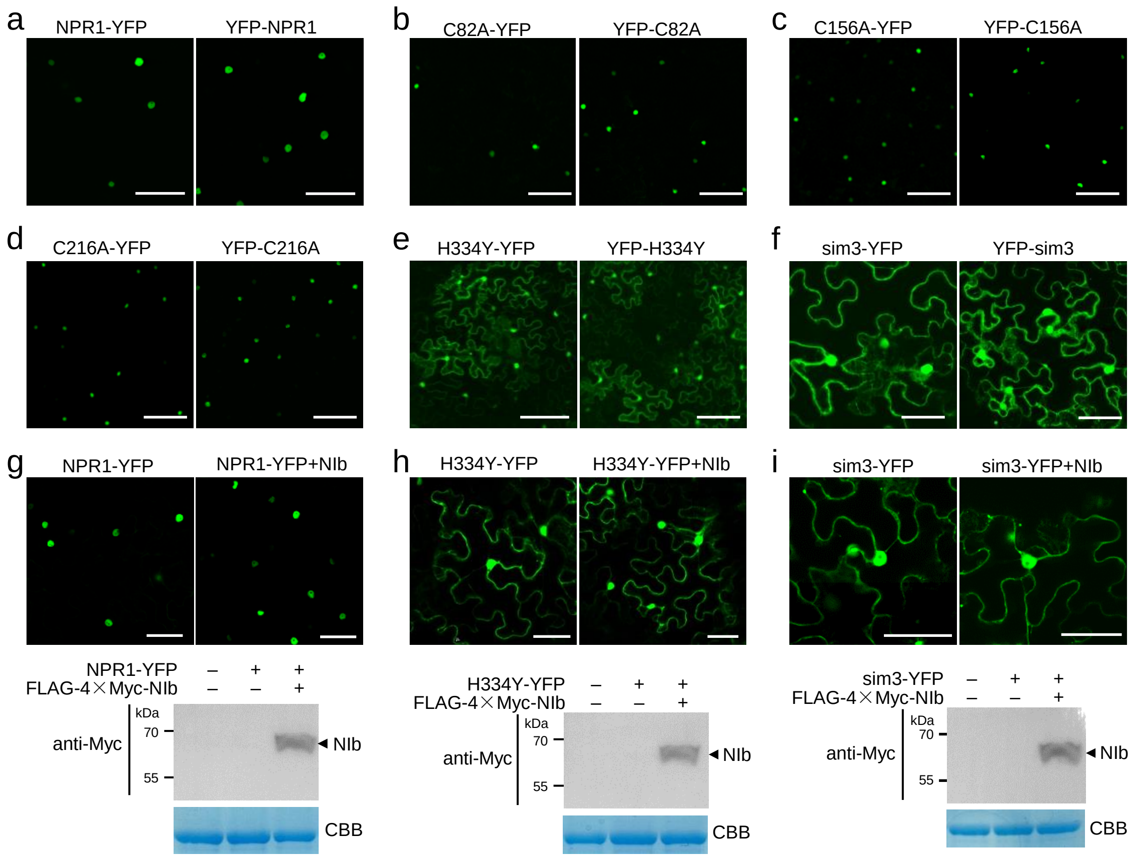

Supplementary Fig. 4 NIb does not affect the subcellular localization of NPR1 and its mutants. a-f Confocal microscopic photos of *N. benthamiana* epidermal cells expressing N-terminal and C-terminal YFP-tagged NPR1 (**a**), C82A (**b**), C156A (**c**), C216A (**d**), H334Y (**e**), sim3 (**f**). **g-i** Effects of NIb on the subcellular localization of NPR1 (**g**), H334Y (**h**) and sim3 (**i**) in *N. benthamiana* epidermal cells. The micrographs were taken at 2 dpi with the same settings; scale bar, 50 μm. Results of immunoblot with anti-Myc antibodies are showing at the bottom panel to indicate the expression of NIb. All experiments were independently repeated three times with similar results.

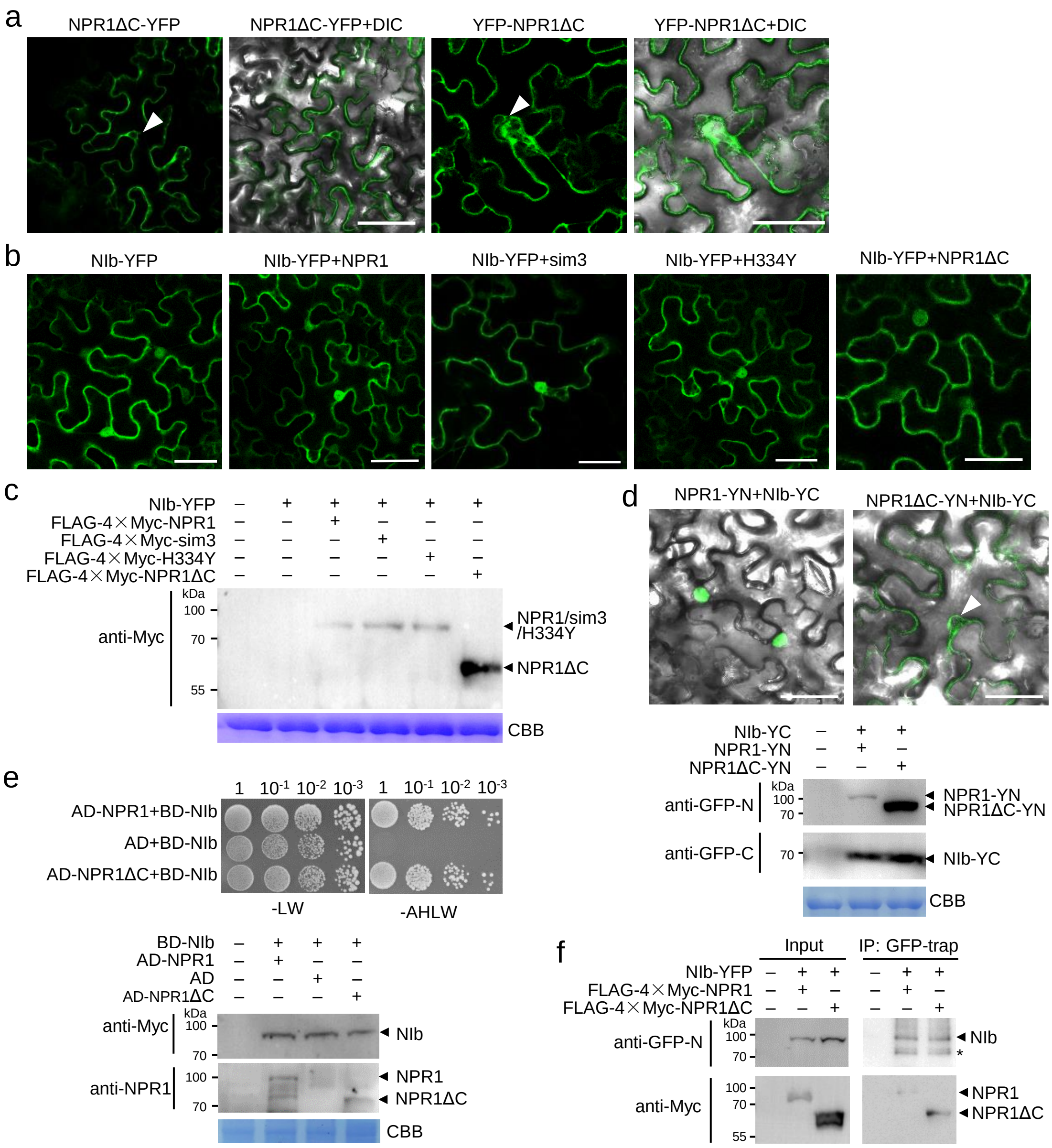

Supplementary Fig. 5 The subcellular localization of NIb is not influenced by NPR1 and its mutants. **a** Confocal microscopic photos of *N. benthamiana* epidermal cells expressing N-terminal and C-terminal YFP-tagged NPR1ΔC; differential interference contrast channel (DIC) was included to indicate cell contours; white arrowheads indicate nuclei; scale bar, 50 μm. **b** Confocal microscopic photos of *N. benthamiana* epidermal cells expressing NIb-YFP and FLAG-4×Myc-tagged NPR1, sim3, H334Y or NPR1ΔC. The micrographs were taken at 2 dpi with the same settings; scale bar, 50 μm. **c** Immunoblots for the expression of NPR1, sim3 and H334Y in penal **b** with anti-Myc antibodies. **d** Confocal microscopic photographs of *N. benthamiana* epidermal cells expressing NIb-YC and NPR1-YN or NPR1ΔC-YN at 2 dpi; scale bar, 50 μm; immunoblot results are showing at the bottom panel to indicate the expression of each protein. **e** Growth of serially diluted yeast cells that were transformed with BD-NIb and AD, AD-NPR1 or AD-NPR1ΔC on selective medium; the expression of each protein was analyzed by immunoblot (bottom panel). **f** Co-IP assay to test the interaction between NIb and NPR1 or NPR1ΔC; proteins were expressed in *N. benthamiana* leaves by agroinfiltration, immunoprecipitated with GFP-trap agarose at 2 dpi, and analyzed by anti-GFP N-terminal (anti-GFP-N) or anti-Myc antibodies; the nonspecific band is indicated by an asterisk. All experiments were independently repeated twice with similar results.

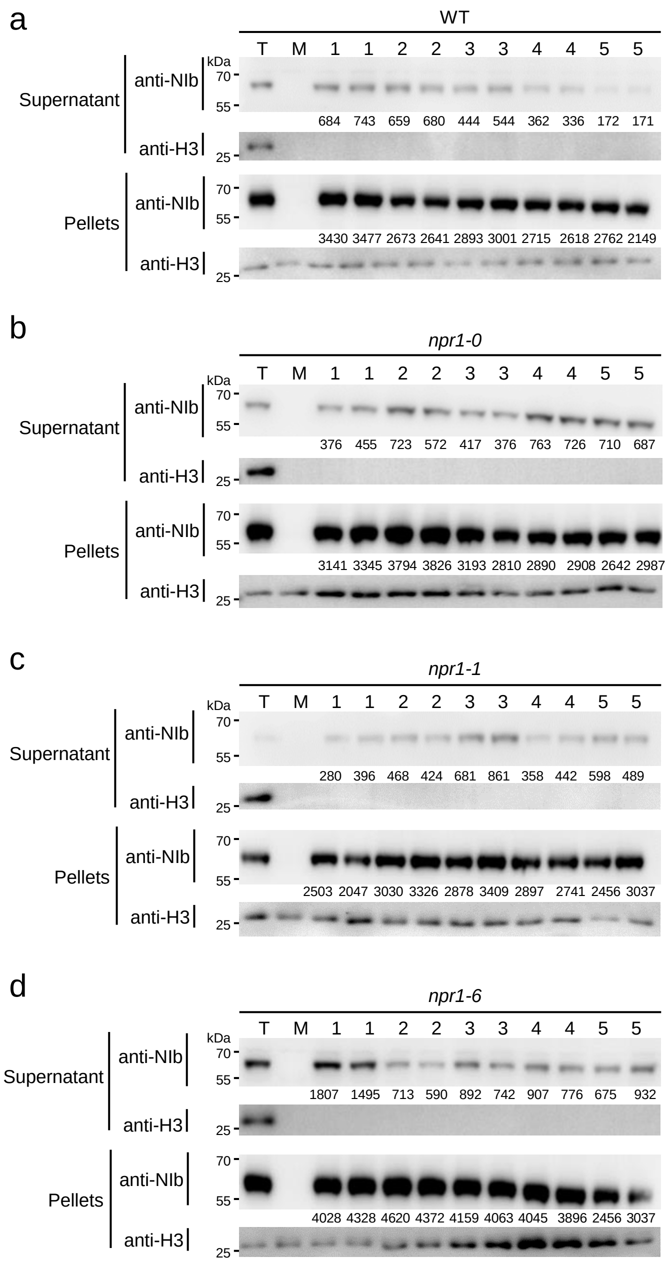

Supplementary Fig. 6 NPR1 does not affect the nucleocytoplasmic portioning of NIb. a-d Immunoblots for NIb in the supernatant (cytoplasmic fraction) and pellets (nuclear fraction) of cell lysates from TuMV-infected WT (**a**), *npr1-0* (**b**), *npr1-1* (**c**), and *npr1-6* (**d**) seedlings with anti-NIb antibodies. Equal amounts of supernatant and pellets were loaded for immunoblotting. Histone 3 (H3) was detected with anti-H3 antibodies to indicate whether the nucleus was completely pelleted. T, total protein of TuMV-infected WT seedlings; M, total protein of mock-treated WT seedlings. Lines 1-5 indicate five independent seedling repeats.

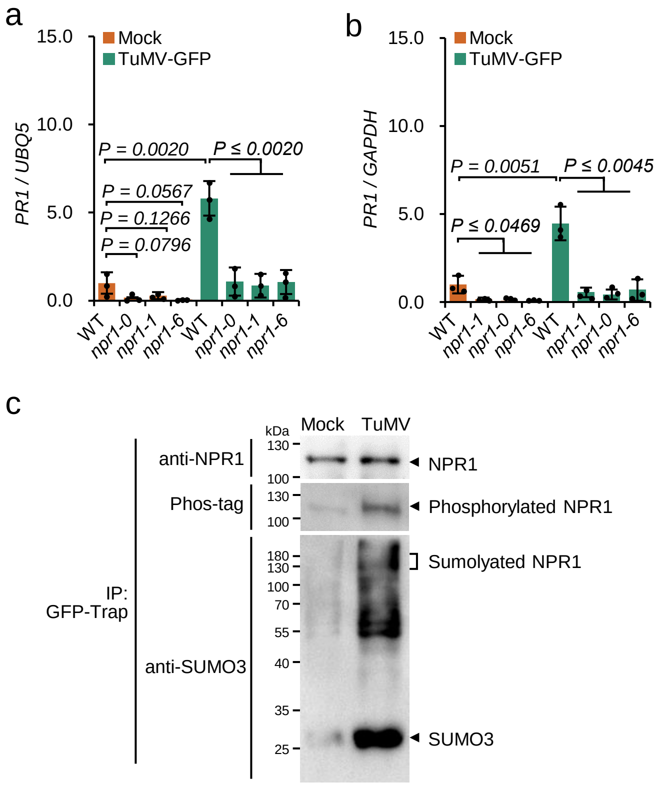

Supplementary Fig. 7 NPR1 is required for inducing the expression of *PR* genes during TuMV infection. a, **b** Bar charts showing the expression of *PR1* in mock- or TuMV-infected WT plants and *npr1* mutants at 48 hpi with *UBQ5* (**a**) and *GAPDH* (**b**) as the internal controls (n = 3). RT–qPCR experiments were performed with the same RNA samples as in Fig. 2f. The expression of *PR1* in mock-infected WT plants was normalized to 1. **c** Immunoblot analysis of the phosphorylation and sumoylation of NPR1 after TuMV infection. NPR1 was immunoprecipitated with GFP-trap agarose at 14 dpi and then analyzed by anti-NPR1 antibodies (top panel), treated with Phos-tag and then detected by streptavidin-HRP (middle panel), or detected by Anti-SUMO3 polyclonal antibodies (bottom panel). The experiment was independently repeated twice with similar results. Data are presented as mean values ± SD. Statistical analyses were performed using Two-tailed Student's *t*-test. Source data are provided as a Source Data file.

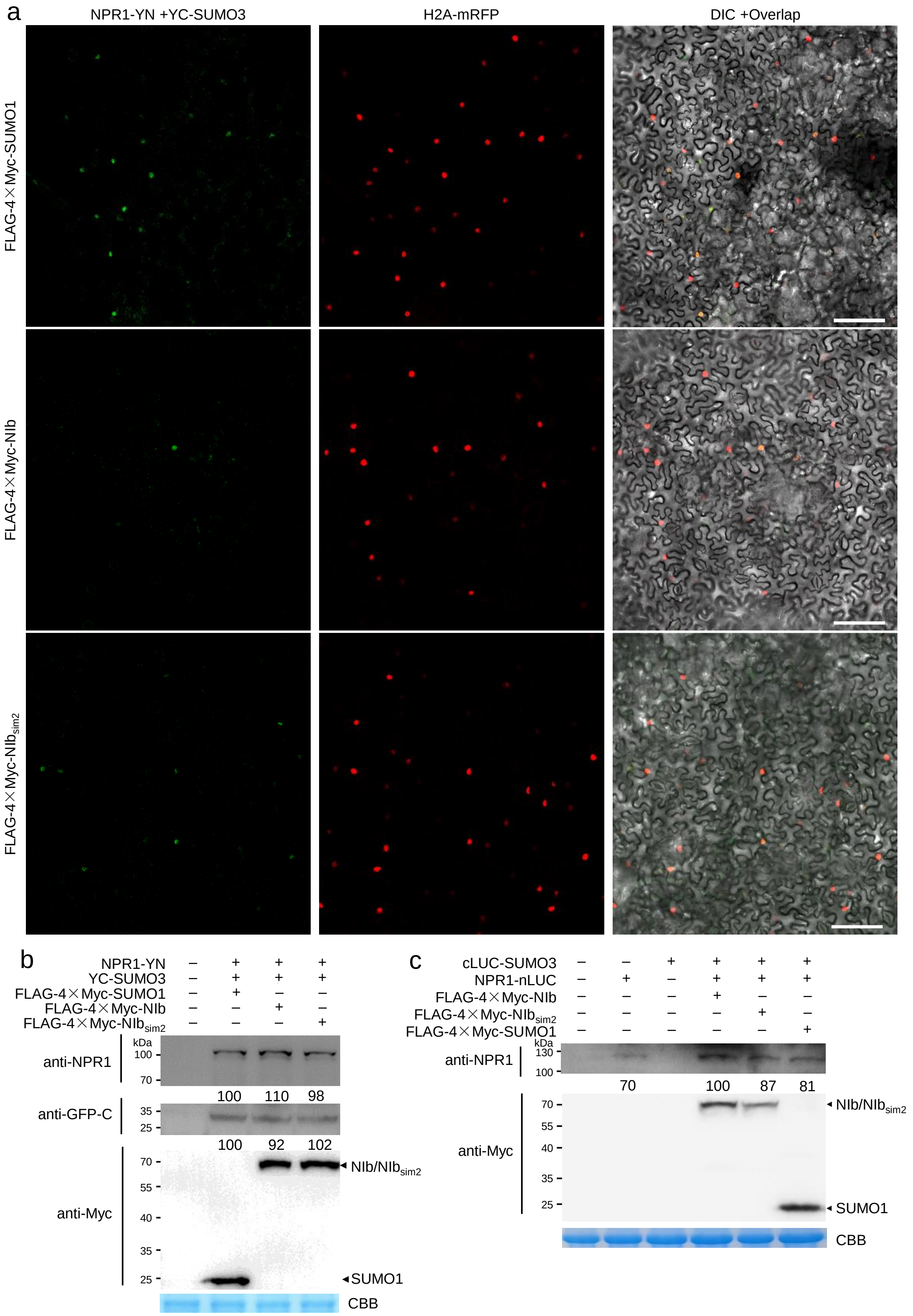

Supplementary Fig. 8 SUMO3 or SUMO3-mediated sumoylation is not required by NIb for interaction with NPR1. a Representative micrographs of *N. benthamiana* epidermal cells coexpressing NPR1-YN + YC-SUMO3 plus SUMO1, NIb, or NIb$_{sim2}$ at 60 hpi. H2-mRFP, mRFP-tagged histone H2A.

All micrographs were taken under exactly the same conditions. Scale bars, 100 μm. **b** Immunoblots for the protein expression of the competitive BiFC assay in Fig. 3a. NPR1-YN, YC-SUMO3, and FLAG-4×Myc-tagged SUMO1, NIb, or NIb_{sim2} were detected with anti-NPR1, anti-GFP-C, and anti-Myc antibodies, respectively. **c** Immunoblots for the protein expression of the split-luciferase assay in Fig. 3b. NPR1-nLUC and FLAG-4×Myc-tagged SUMO1, NIb, and NIb_{sim2} were detected with anti-NPR1 and anti-Myc antibodies, respectively. All experiments were independently repeated twice with similar results.

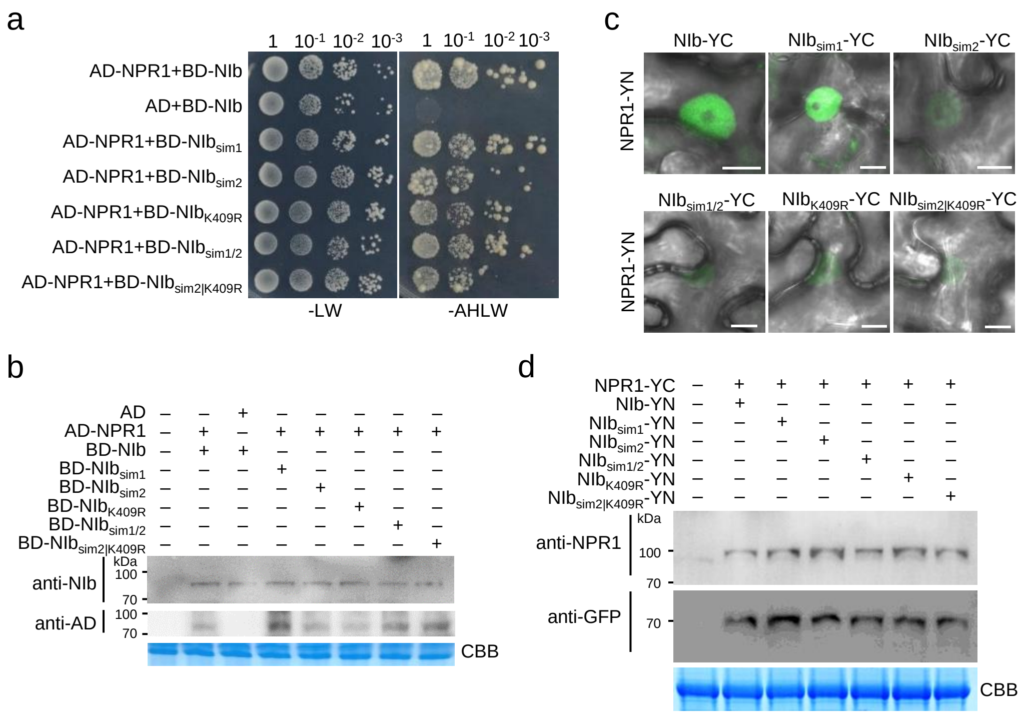

Supplementary Fig. 9 Sumoylation increases the affinity of NIb to NPR1. a Growth of serially diluted yeast cells transformed with the indicated plasmids on selective medium. **b** Immunoblots for the expression of AD-NPR1 and BD-tagged NIb or its mutants of the Y2H assay in panel **a** with anti-AD and anti-NIb antibodies, respectively. The experiment was independently repeated twice with similar results. **c** Confocal microscopic photographs of *N. benthamiana* epidermal cells expressing NPR1-YN and YC-tagged NIb or its mutants at 2 dpi. Scale bar, 20 μm. The experiment was independently repeated three times with similar results. **d** Immunoblots for the expression of NPR1-YC and YN-tagged NIb or its mutants of the BiFC assay in panel **c** with anti-NPR1 and anti-GFP antibodies, respectively. The experiment was independently repeated three times with similar results.

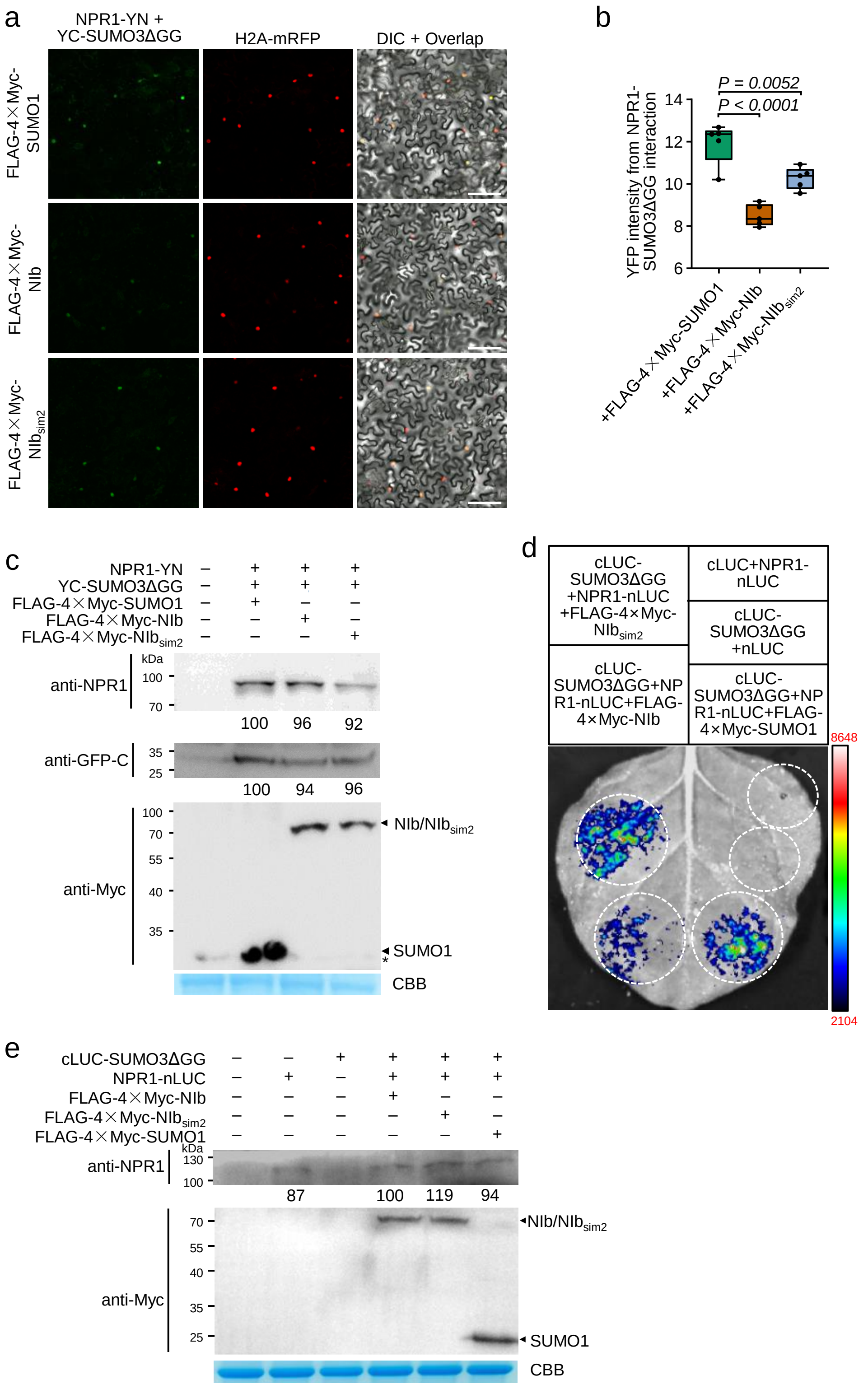

Supplementary Fig. 10 NIb disrupts the SUMO3–NPR1 interaction. a Representative micrographs of *N. benthamiana* epidermal cells coexpressing NPR1-YN + YC-SUMO3ΔGG plus SUMO1, NIb, or NIb_{sim2} at 60 hpi. Scale bars, 100 μm. **b** Box and whisker plot with individual data points comparing the average nuclear YFP signal intensities of the interaction between NPR1-YN and YC-SUMO3ΔGG in the presence of SUMO1, NIb, or NIb_{sim2}. The data are the mean of the nuclear signal intensity of 5 micrographs. The whisker indicates minimum and maximum, and the box indicates the 25th and 75th percentiles (edges of

the box), and median (center line). **c** Immunoblot for NPR1-YN, YC-SUMO3ΔGG, and FLAG-4×Myc-tagged SUMO1, NIb, or NIb_{sim2} of the competitive BiFC assay in panel **a** with anti-NPR1, anti-GFP-C, and anti-Myc antibodies, respectively. The experiment was independently repeated three times with similar results. **d** Influence of SUMO1, NIb, or NIb_{sim2} on the luciferase activity in *N. benthamiana* leaf tissues expressing cLUC or cLUC-SUMO3ΔGG and NPR1-nLUC or nLUC plus FLAG-4×Myc-tagged SUMO1, NIb or NIb_{sim2}. **e** Immunoblots for NPR1-nLUC and FLAG-4×Myc-tagged SUMO1, NIb, or NIb_{sim2} of the competitive split-luciferase assay in panel **d** with anti-NPR1 and anti-Myc antibodies, respectively. The experiment was independently repeated twice with similar results.

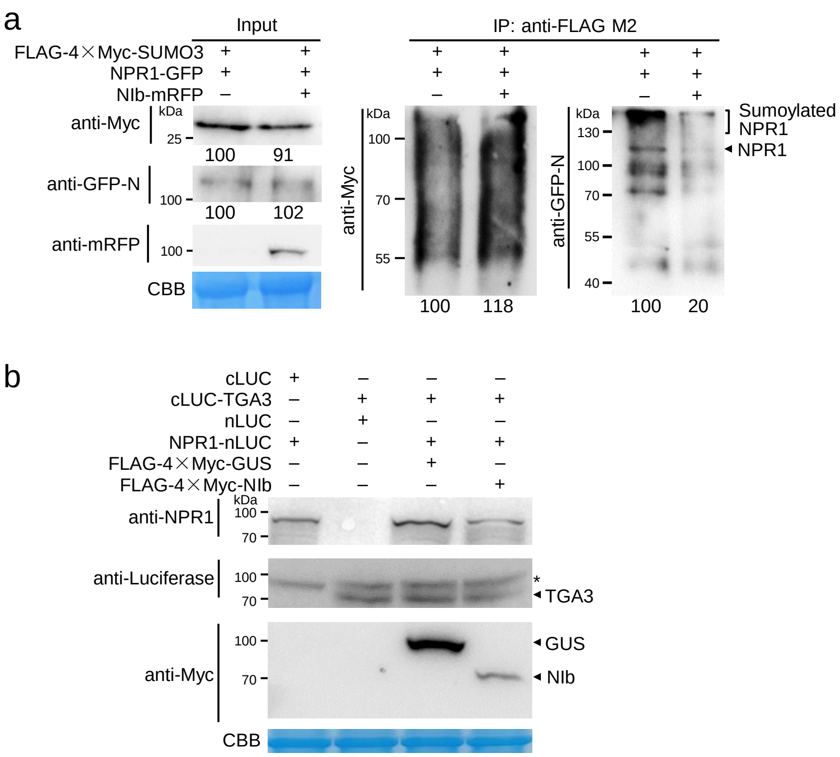

Supplementary Fig. 11 NIb inhibits NPR1 sumoylation. a Analysis of NPR1 sumoylation by immunoblotting. Proteins were expressed in *N. benthamiana* epidermal cells, immunoprecipitated by anti-FLAG M2 affinity gel at 2 dpi and then analyzed with anti-Myc (middle panel) or anti-GFP-N (right panel) antibodies. The expression levels of NIb-mRFP, NPR1-GFP and FLAG-4×Myc-SUMO3 were also analyzed by Western blotting before immunoprecipitation using anti-mRFP, anti-GFP-N, and anti-Myc antibodies, respectively (left panel). **b** Immunoblots for NPR1-nLUC, cLUC-TGA3, and FLAG-4×Myc-tagged GUS or NIb of the competitive split-luciferase assay in Fig. 3d with anti-NPR1, anti-luciferase, and anti-Myc antibodies, respectively. The asterisk indicates a nonspecific band. All experiments were independently repeated three times with similar results.

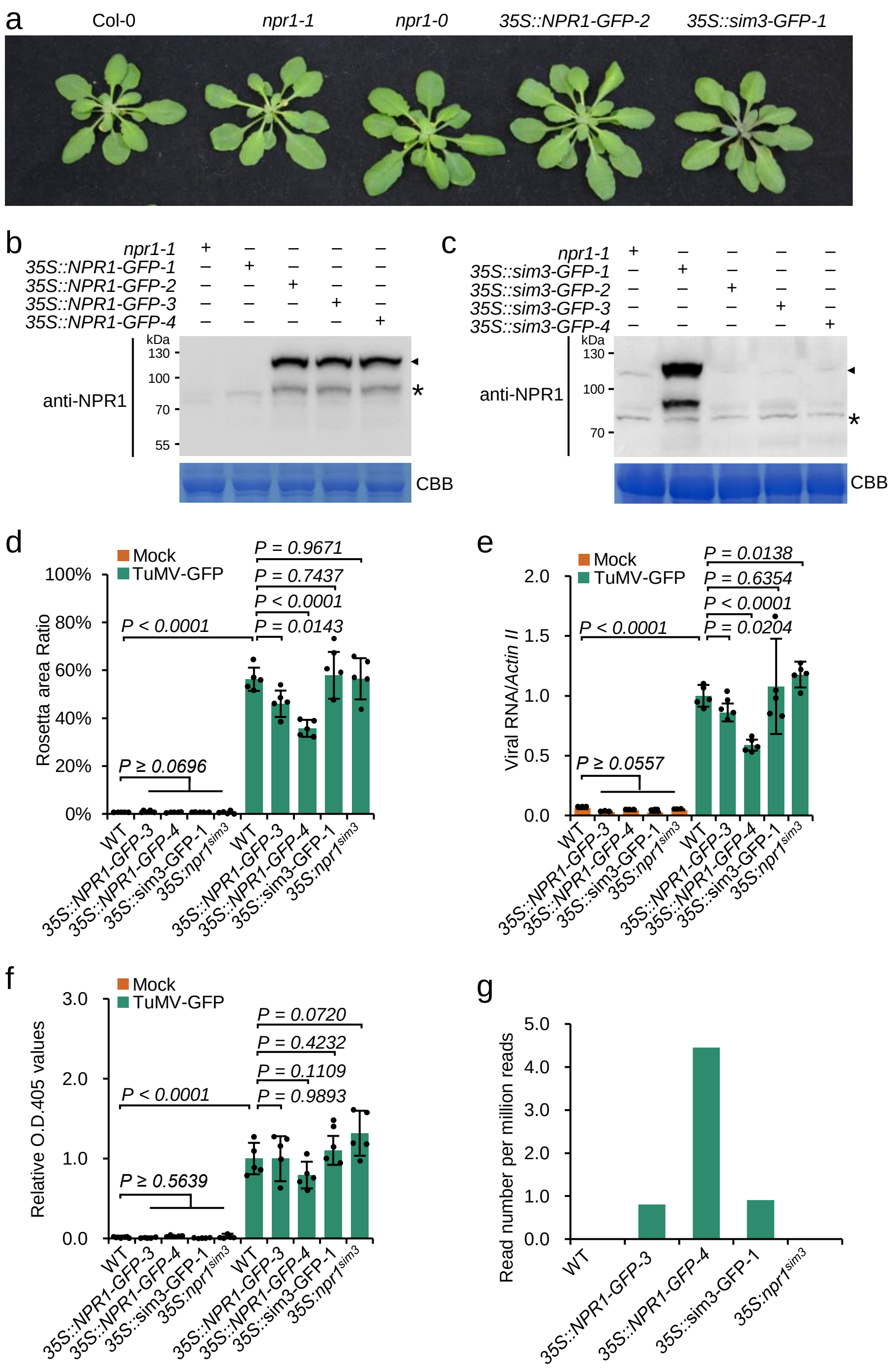

Supplementary Fig. 12 NIb affects NPR1 sumoylation. **a** Phenotypes of three-week-old WT, *npr1-1*, *npr1-0*, *35S::NPR1-GFP-2,* and *35S::sim3-GFP-1* seedlings. **b**, **c** Immunoblots for NPR1-GFP (**b**) and sim3-GFP (**c**) in one-week-old *35S::NPR1-GFP* and *35S::NPR1^{sim3}-GFP* seedlings with anti-NPR1 antibodies. Asterisks indicate nonspecific bands. **d** Bar chart of the ratio of TuMV-infected leaf area to the total leaf area at 14 dpi (n = 5). **e** Bar chart showing the accumulation of the virus genome in WT and transgenic plants at 18 dpi (n = 5). The viral RNA in TuMV-infected WT plants was normalized to 1. **f** Bar chart showing the accumulation of TuMV virions in WT and transgenic plants at 18 dpi (n = 5). The ELISA readings were taken after 1 h of substrate hydrolysis. The O.D.450 value of TuMV-infected WT plants was normalized to 1. **g** Bar chart showing GFP-derived sRNA in WT, *35S::NPR1-GFP-4*, *35S::NPR1-GFP-3*,

35S::sim3-GFP-1, and *35S:npr1^{sim3}* seedlings. Data are presented as mean values ± SD. Statistical analyses were performed using Two-tailed Student's *t*-test. Source data are provided as a Source Data file.

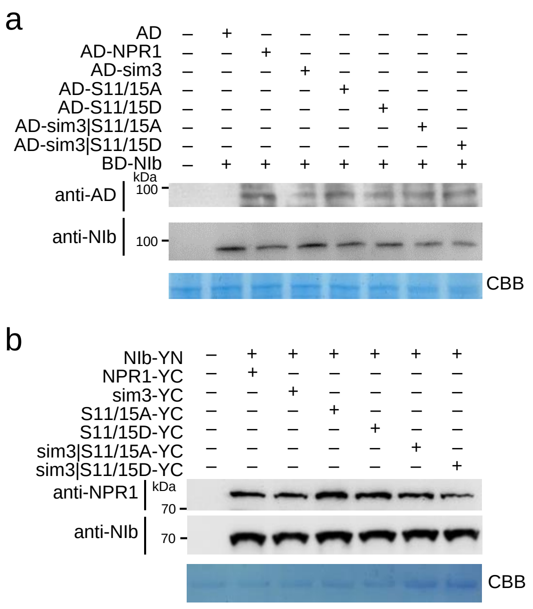

Supplementary Fig. 13 Phosphorylation increases the affinity of NIb to NPR1. a Immunoblots for BD-NIb and AD-tagged NPR1 or its mutants of the Y2H assay in Fig. 4a with anti-NIb and anti-AD antibodies, respectively. **b** Immunoblots for NIb-YN and YC-tagged NPR1 or its mutants of the BiFC assay in Fig. 4b with anti-NIb and anti-NPR1 antibodies, respectively.

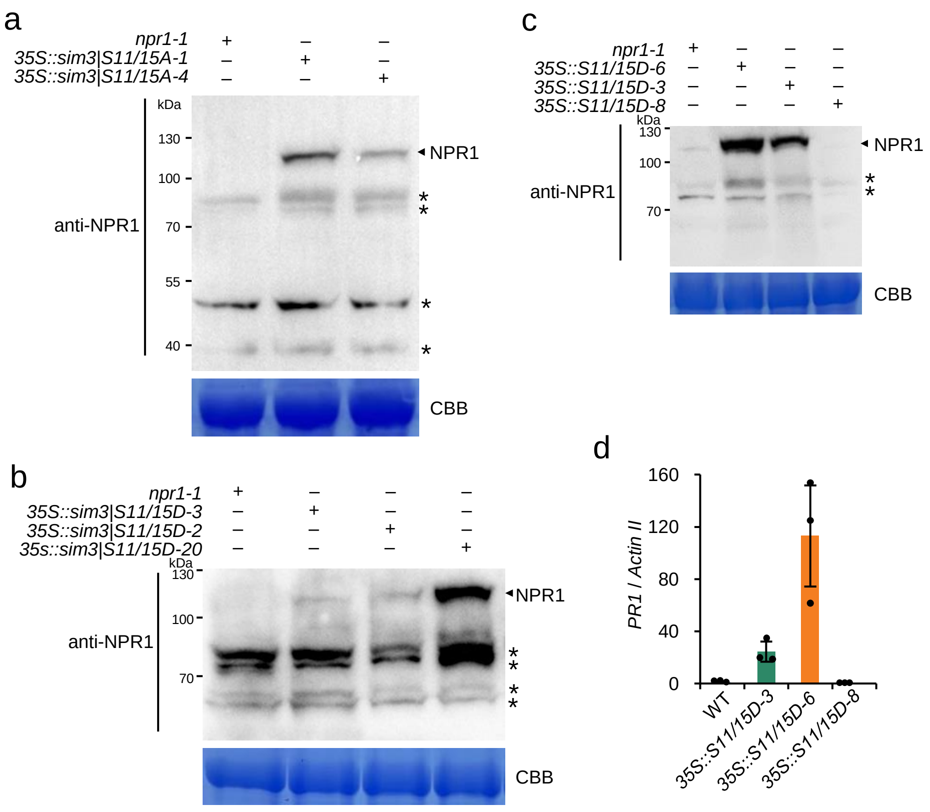

Supplementary Fig. 14 Phosphorylation at Ser11/Ser15 induces the expression of *PR* genes. a–c Immunoblots for protein expression in the seedlings of *35S::sim3|S11/15A-GFP* (**a**), *35S::sim3|S11/15D-GFP* (**b**), and *35S::S11/15D-GFP* (**c**) with anti-NPR1 antibodies. Asterisks indicate nonspecific bands. **d** Bar chart showing the expression level of *PR1* in the WT and three independent *35S::S11/15D-GFP* lines (n = 3). *Actin II* was used as the internal control in the RT–qPCR. The expression of *PR1* in mock-infected WT plants was normalized to 1. Data are presented as mean values ± SD. Source data are provided as a Source Data file.

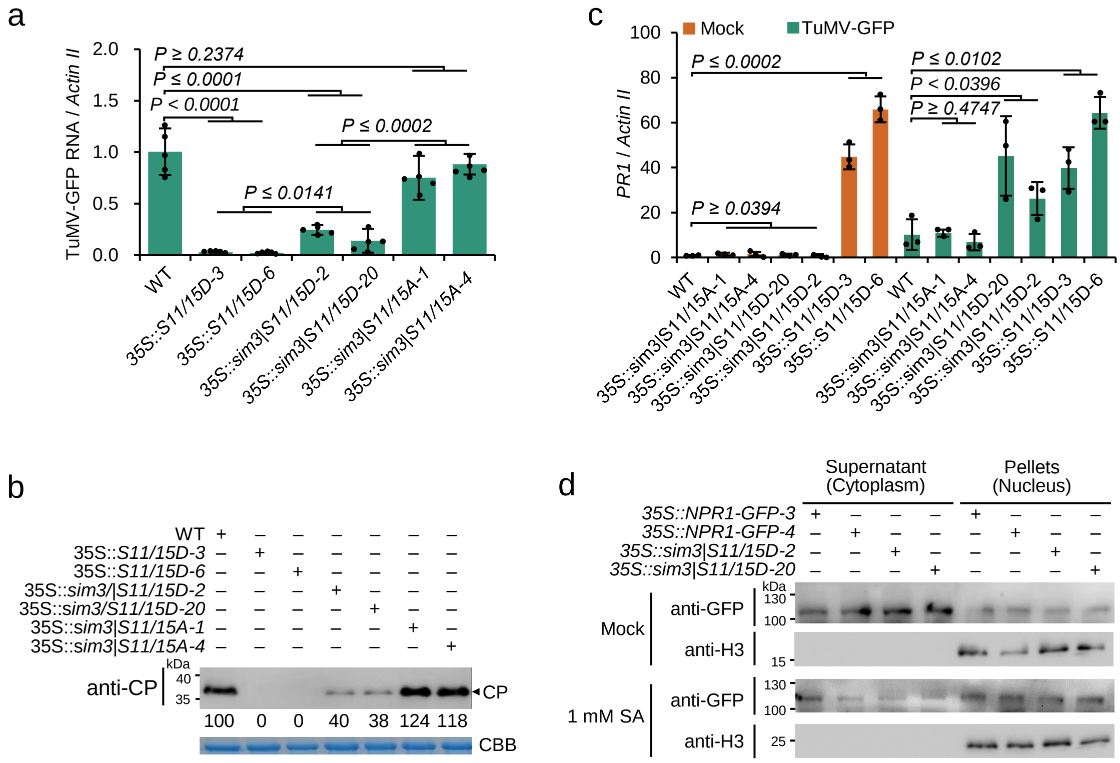

Supplementary Fig. 15 Proliferation of TuMV is attenuated in phosphorylation mimic NPR1 mutants. a Bar chart showing the accumulation of the virus genome in WT and transgenic plants at 18 dpi (n = 5). **b** Immunoblot analysis of the accumulation of viral CP in WT and transgenic plants at 18 dpi with anti-CP antibodies. The number under each lane indicate the relative intensity of the band. **c** Bar chart of relative *PR1* expression level in mock or virus-infected WT, *35S::sim3|S11/15A-GFP*, *35S::sim3|S11/15D-GFP*, and *35S:S11/15D-GFP* plants at 48 hpi (n = 3). The expression of *PR1* in mock WT plants was normalized to 1. **d** Immunoblots for sim3|S11/15D-GFP in the supernatant (cytoplasmic fraction) and pellets (nuclear fraction) of cell lysates from mock or SA treated seedlings of *35S::sim3|S11/15D-GFP* with anti-GFP antibodies. Equal amounts of supernatant and pellets were loaded for immunoblotting. Histone 3 (H3) was detected with anti-H3 antibodies to indicate whether the nucleus was completely pelleted. Data are presented as mean values ± SD. Statistical analyses were performed using Two-tailed Student's *t*-test. Source data are provided as a Source Data file.

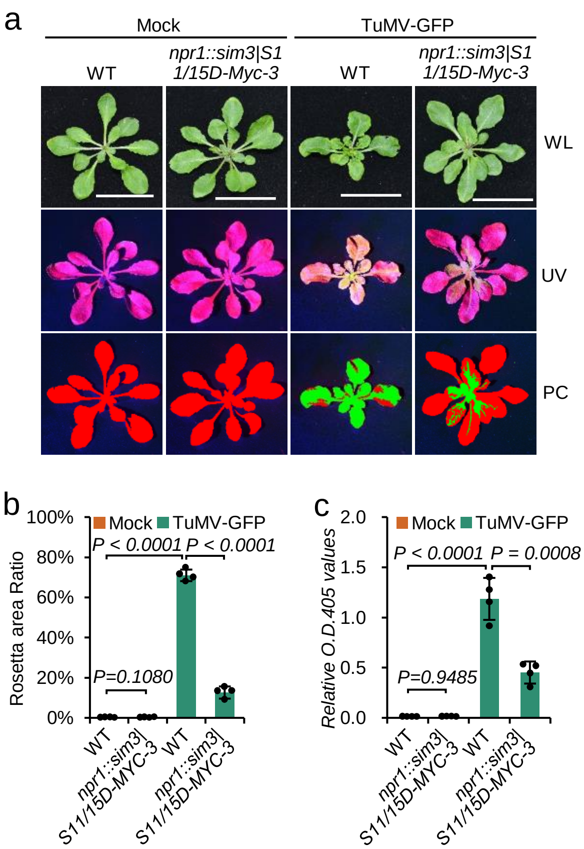

Supplementary Fig. 16 The infectivity of TuMV-GFP is attenuated in transgenic plants expressing sim3|S11/15D. a Phenotypes of mock- or virus-inoculated WT and transgenic *npr1::sim3|S11/15D-MYC-3* at 18 dpi. **b** Bar plot showing the ratio of the infected area to the total area of WT and transgenic *npr1::sim3|S11/15D-MYC-3* plants at 18 dpi (n = 4). **c** Bar plot showing the accumulation of virions in WT and *npr1::sim3|S11/15D-MYC-3* plants at 18 dpi. ELISA readings were taken after 1 h of substrate hydrolysis (n = 4). Data are presented as mean values ± SD. Statistical analyses were performed using Two-tailed Student's *t*-test. Source data are provided as a Source Data file.

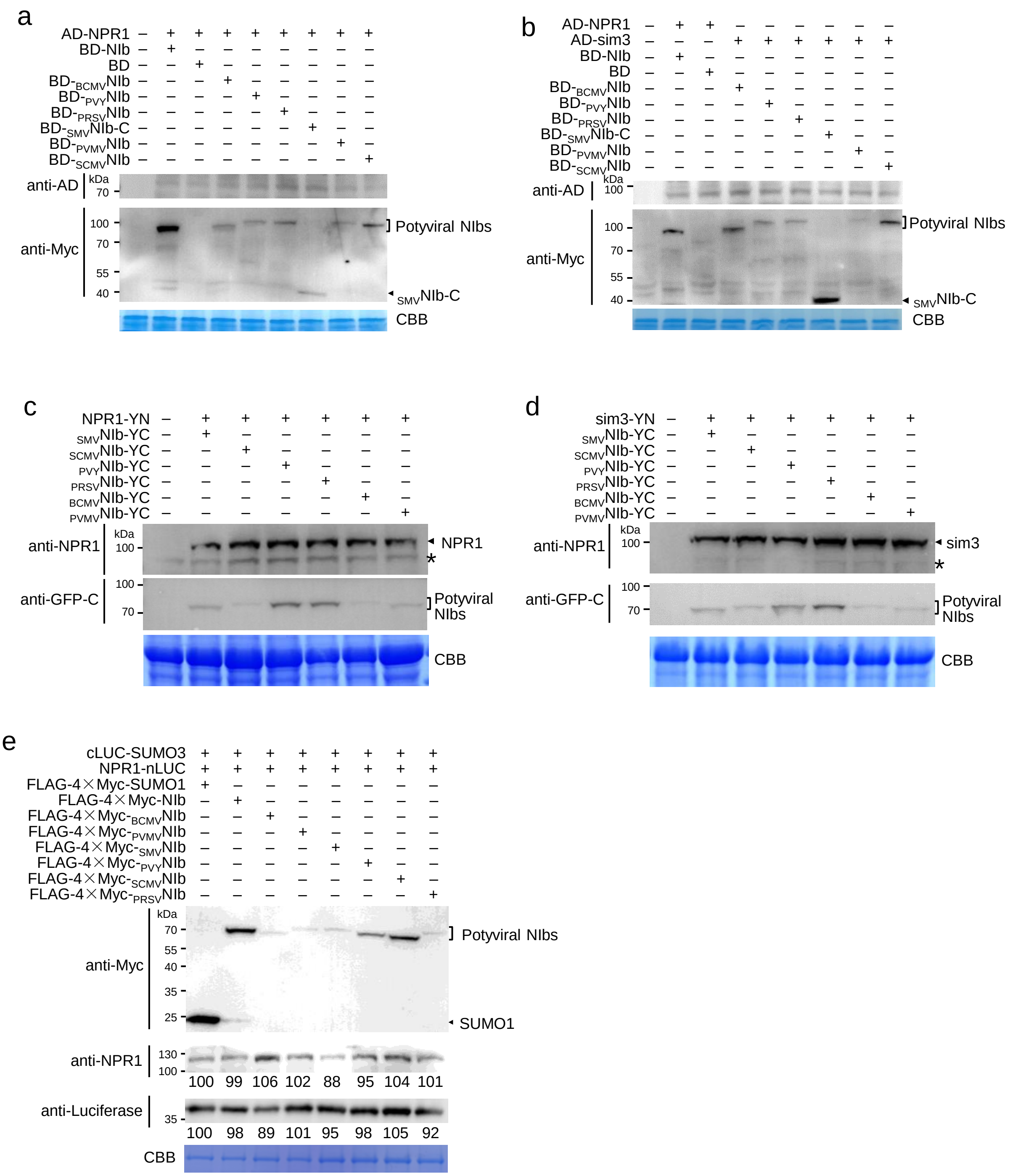

Supplementary Fig. 17 NIb proteins of different potyviruses interact with NPR1. **a**, **b** Immunoblots for the expression of recombinant proteins of Y2H assays in Fig. 5a (**a**) and 5b (**b**) with anti-Myc and anti-AD antibodies. **c**, **d** Immunoblots for the expression of recombinant proteins of BiFC assays in Fig. 5c (**c**) and 5d (**d**). YC-tagged potyviral NIb proteins and NPR1-YN or sim3-YN were detected with anti-GFP C-terminal (GFP-C) and anti-NPR1 antibodies, respectively. The asterisk indicates a nonspecific band detected with anti-NPR1 antibodies. **e** Immunoblots for the expression of NPR1-nLUC (anti-NPR1), cLUC-SUMO3 (anti-Luciferase), and FLAG-4×Myc-tagged SUMO1 or NIb proteins (anti-Myc) of split-luciferase assay in Fig. 5e. All experiments were independently repeated three times with similar results.

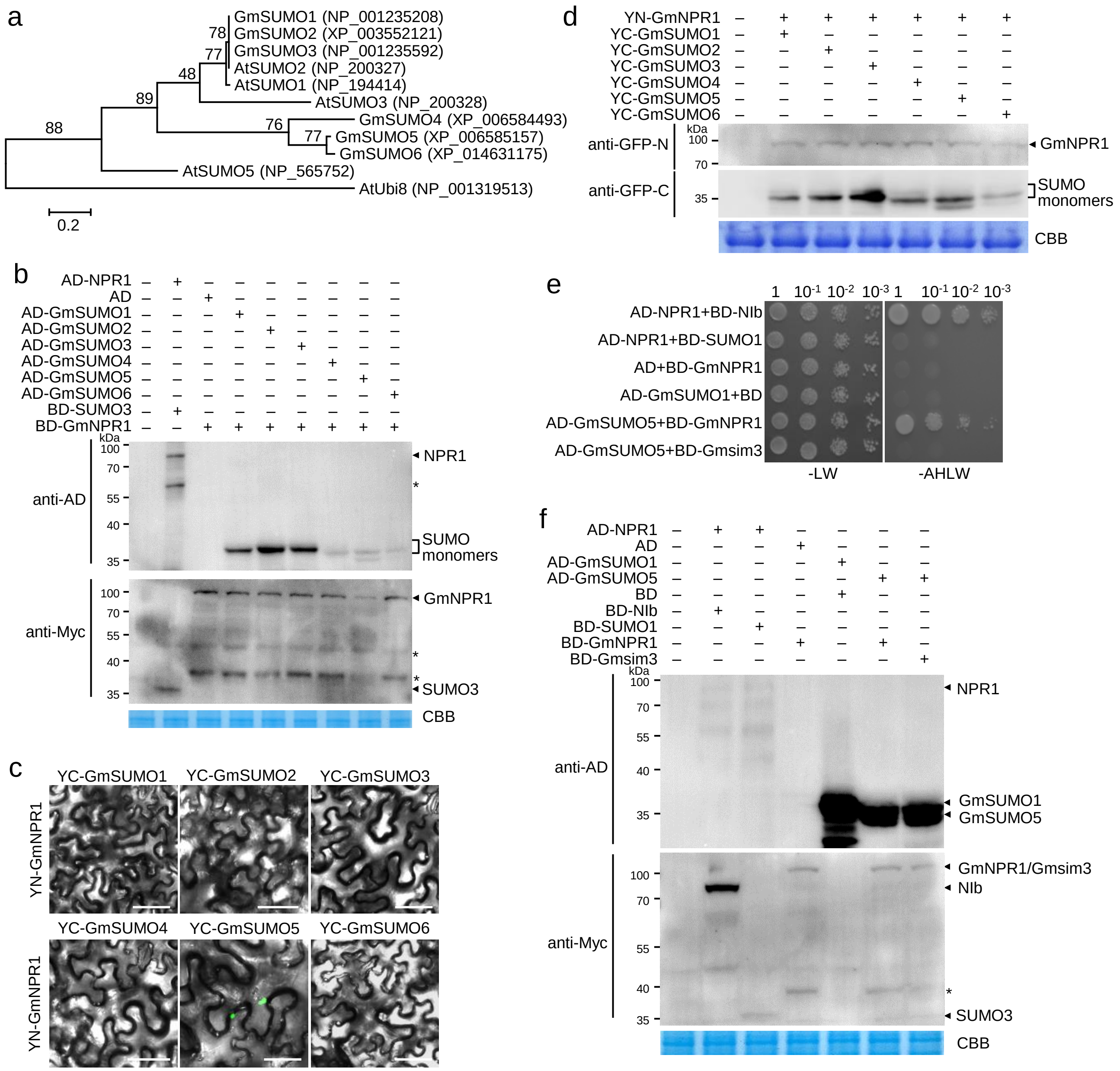

Supplementary Fig. 18 Soybean NPR1 is sumoylated by GmSUMO5. a Phylogeny of Arabidopsis and soybean SUMO paralogs. The phylogenetic tree was constructed using MEGA X software with the neighbor-joining method. The numbers on the branches represent the percentage values of 1000 bootstrap replicates. The GenBank accession numbers are indicated in brackets. **b** Immunoblots for BD-GmNPR1 and AD-tagged NPR1 or soybean SUMO homologs of the Y2H assay in Fig. 6b with anti-Myc and anti-AD antibodies, respectively. **c** Confocal microscopic photos of *N. benthamiana* epidermal cells expressing YN-GmNPR1 and YC-tagged GmSUMO homologs at 2 dpi. All micrographs were taken with the same settings. Bars = 50 μM. **d** Immunoblots for the expression of YN-GmNPR1 and YC-tagged GmSUMO1-SUMO6 of the BiFC assay in panel **c** with anti-GFP-N and anti-GFP-C antibodies, respectively. **e** Assessment of the interaction between GmNPR1 and its SIM3 mutant (Gmsim3) by Y2H assay. **f** Immunoblots for the expression of recombinant proteins of the Y2H assay in penal **e** with anti-AD and anti-Myc antibodies. All experiment were independently repeated three times with similar results.

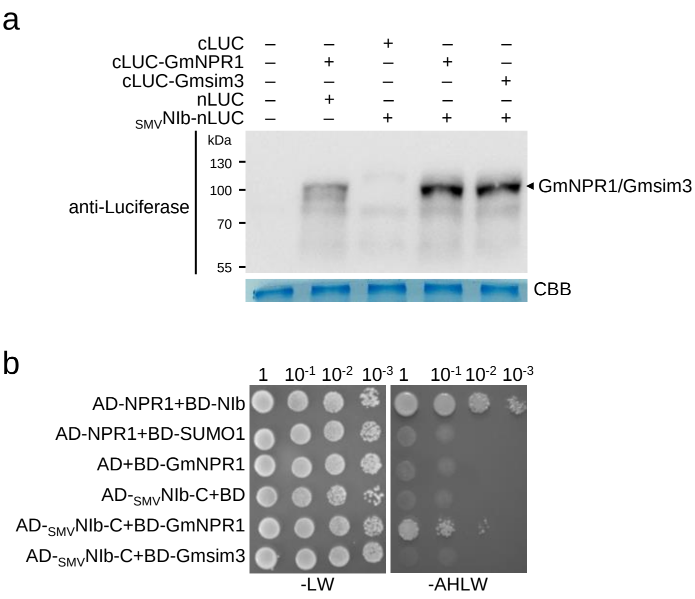

Supplementary Fig. 19 SMV-encoded NIb targets SIM3 of GmNPR1. a Immunoblots for the expression of recombinant proteins of split-luciferase assay in Fig. 6c with anti-Luciferase antibodies. The experiment was independently repeated twice with similar results. **b** Growth of serially diluted yeast cells that were transformed with the indicated plasmids on selective medium.
