# Supplementary material for: A plant RNA virus inhibits NPR1 sumoylation and subverts NPR1-mediated plant immunity
Source: Nat Commun. 2023 Jun 16;14:3580. doi: 10.1038/s41467-023-39254-2 (PMC10275998; doi:10.1038/s41467-023-39254-2)
Supplement: Supplementary file 4 — Source data [file 41467_2023_39254_MOESM4_ESM.zip › Anti-CP.pdf]

ABclonal Technology®

尊重 · 专注 · 服务 · 使命

武汉爱博泰克生物科技有限公司
ABclonal Biotechnology co.,Ltd

多克隆抗体技术服务
项目报告

地址：武汉市东湖高新技术开发区高新二路 388 号武汉国际生物
医药企业加速器 7 栋 4 层
网址：abclonal.com.cn
电话：400-999-6126

项目编号：WG-04332D

1、项目信息

**客户单位**：东北农业大学	**客户姓名**：刘佳慧
**Email**：943092135@qq.com	**联系电话**：13136659817
**项目启动时间**：2020/07/15	**项目结束时间**：
**物种名称**：turnip mosaic virus	**基因名称**：cp
**蛋白大小**：	**NCBI 登录号**：
**制备路线**：多抗蛋白路线	**纯化方式**：抗原亲和纯化
**兔号与抗体浓度**：E15946　1.92mg/mL	**兔号与抗体浓度**：E15947　2.57mg/mL
**项目概述**：<br>用客户提供的含 cp 序列质粒为模板，选择 1-289aa 构建到 pGEX4T-AB1 载体，原核表达免疫原，免疫两只实验级日本大耳白兔，牺牲兔子后，最终提供亲和纯化后的抗体。	

2、项目报告内容

2.1 抗原制备

2.1.1 表达质粒构建

1） **客户模板鉴定**

结果分析：

以客户提供的含 cp 序列质粒为模板，尝试进行 PCR。

2） **抗原片段大小**

1-289aa

3） **表达载体说明**

pGEX-4T-AB1：GST-Tag（211aa）、His-Tag(6aa)，约 33KD

4） **克隆起止时间**

2020/7/15-2020/7/28:1-289aa 区域成功克隆至 pGEX-4T-AB1 载体

5） **抗原制备 PCR 图片**

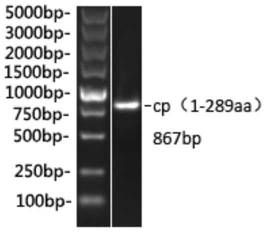

结果分析：

cp（1-289aa）PCR 产物电泳鉴定大小正确，成功克隆到 pGEX-4T-AB1 载体上，并测序鉴定正确，转交表达。

2.1.2 抗原蛋白制备

1） 免疫用蛋白

表达诱导条件

培养到 OD600nm 0.5-0.6 加入 0.8mM IPTG 37℃诱导 4 小时

表达菌株

E. coli Rosetta

表达起止时间

2020/07/28-2020/08/05

少量表达鉴定

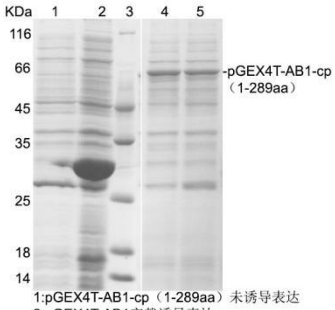

1:pGEX4T-AB1-cp（1-289aa）未诱导表达
2:pGEX4T-AB1空载诱导表达
3:Marker
4:pGEX4T-AB1-cp（1-289aa）诱导表达
5:pGEX4T-AB1-cp（1-289aa）诱导表达

结果分析：

小规模表达测试，目的蛋白有表达，大小在 60KD。

破菌纯化后鉴定

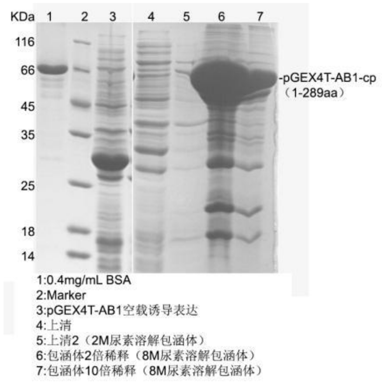

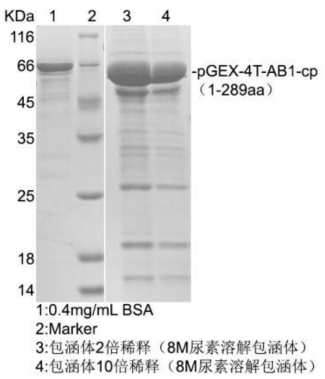

结果分析：

1. pGEX-4T-AB1-cp（1-289aa）表达在包涵体中。

2. 包涵体蛋白浓度为 15mg/mL，纯度达到免疫要求，转交免疫。

2.2 免疫流程

免疫次数	免疫周期	免疫时间	免疫剂量	免疫佐剂	免疫动物状态
第一次免疫	1 天	2020/8/7	0.3mg	完全弗氏佐剂	良好
第二次免疫	12 天	2020/8/19	0.15mg	不完全弗氏佐剂	良好
第三次免疫	26 天	2020/9/2	0.15mg	不完全弗氏佐剂	良好
第四次免疫	40 天	2020/9/16	0.15mg	不完全弗氏佐剂	良好
免疫动物采血	52 天	2020/9/28			采血正常

2.3 抗血清 ELISA 检测数据

ELISA 包被：pGEX-4T-AB1-cp（1-289aa）

包被浓度：2ug/mL, 100ul/well, in CB buffer

二抗：Peroxidase-conjugated AffiniPure Goat Anti-Rabbit IgG (H+L)

二抗稀释：1:8000

WG-04332D cp	Blank	Negative Control 1:1K	Negative Control 1:64K	Positive 1:1K	Positive 1:4K	Positive 1:8K	Positive 1:16K	Positive 1:32K	Positive 1:64K	Positive 1:128K	Positive 1:256K	Positive 1:512K
E15946	0.0428	0.0384	0.0364	1.0447	1.0059	0.8967	0.7498	0.5664	0.4067	0.2786	0.1691	0.1358
E15947	0.0557	0.0199	0.0264	1.0323	0.9669	0.9026	0.76	0.5768	0.4228	0.2824	0.1649	0.1319

结果分析：经 ELISA 检测，包被 200ng 抗原时，E15946,E15947 四免血清效价在稀释度为 1：64K 时 OD 值均大于 0.4，血清效价合格。

2.4 抗血清纯化

1） **亲和纯化用蛋白检测**

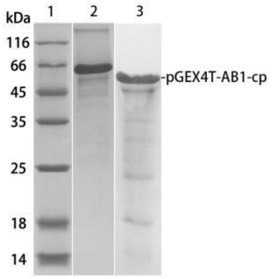

1.Marker
2.0.4mg/mL BSA
3.pGEX4T-AB1-cp diluted in 10 folds

结果分析： 亲和纯化用 pGEX-4T-AB1-cp 蛋白经检测，浓度为 4mg/ml，与破菌纯化后浓度和纯度差异不大，可进行抗原亲和纯化。

2） **抗血清纯化**

抗血清用 pGEX-4T-AB1-cp 蛋白作抗原亲和纯化后，得到浓缩后的抗体：

E15946：浓度 1.92mg/mL

E15947：浓度 2.57mg/mL

2.5 抗原 WB 或内源 WB 检测图

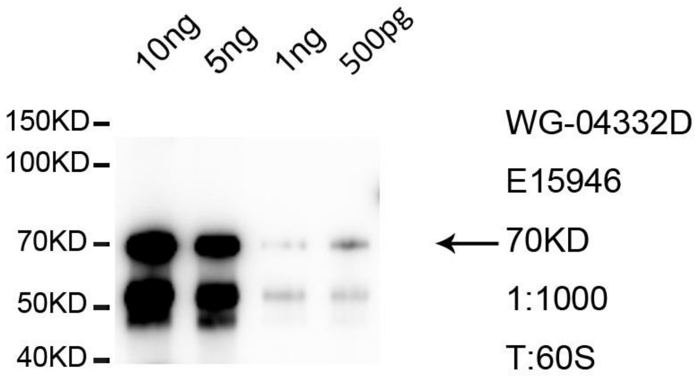

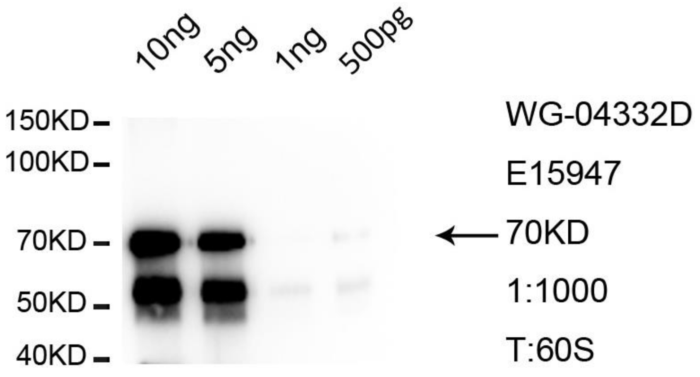

说明： 图中各泳道分别为 10ng，5ng，1ng，500pg 抗原；抗体稀释比例为 1:1000。

结果分析：

1、E15946,E15947 抗体检测抗原条带大小在 70KD 左右；

2、E15946,E15947 抗体 1:1000 稀释可检测到 5ng 抗原；

3、E15946,E15947 抗体浓度正常。

3、抗体使用及保存注意事项

WB 稀释度： 1/500-1/1000

保存条件： -20℃保存，避免反复冻融

缓冲液体系： PBS, 50% glycerol, pH7.3

ABclonal Technology®　　尊重 · 专注 · 服务 · 使命

Wuhan ABclonal Biotechnology Co., Ltd

Project report

Polyclonal antibodies Technical Service

Address: Floor 4, Building 7 of the International Biomedical Enterprise Accelerator, No. 388, Gaoxin Second Road, Wuhan East Lake High-Tech Development Zone, Wuhan
Web：abclonal.com.cn
Tel：400-999-6126

项目编号：WG-04332D

1、Project information

Client employer: Northeast Agricultural University	Client Name: Jiahui Liu
Email：943092135@qq.com	Tel: 13136659817
Project start time: 2020/07/15	Project finish time:
Species name: Turnip mosaic virus	Gene name: CP
Protein size	NCBI Accession no.
Technical route: polyclonal	Method of purification: Antigen-affinity purification
Rabbit ID and antibody concentration: E15946 1.92 mg/ml	Rabbit ID and antibody concentration: E15947 2.57 mg/ml
Project summary：<br>Using the plasmid containing CP, which is provided by client, as the template, the fragment of CP encoding 1-289 aa was inserted into pGEX4T-AB1, the antigen was expressed prokaryotically and was used to immunize two experimental-grade Japanese big-eared white rabbits, after sacrificing the rabbits, the antibodies were affinity purified and finally provided to client.	

2、Project report content

2.1 Antigen preparation

2.1.1 Construction of expression plasmid

1) Identification of client template

Analysis of results

The plasmid containing CP that was provided by client was used as the template for PCR.

2) Antigen fragment size

1-289aa

3) Expression vector specification

pGEX-4T-AB1：GST-Tag(211aa)、His-Tag(6aa)，about 33 KD

4) Start and end time of cloning

2020/7/15-2020/7/28: The region 1-289 aa of CP was successfully cloned into pGEX-4T-AB1 vector.

5) PCR results of antigen preparation

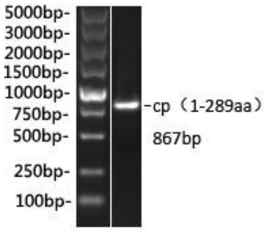

Result Analyses

The size of cp(1-289 aa) PCR product was identified correctly by electrophoresis, and it was successfully cloned into pGEX-4T-AB1 vector, identified correctly by sequencing, and was used for expression.

2.1.2 Preparation of antigen

1) Protein for immunization

Protein induction condition

Culture to O.D.600 nm 0.5-0.6, adding 0.8 mM IPTG to induce at 37℃ for 4 h.

Expression bacteria strain:

E.coli Rosetta

Start and end time of protein expression

2020/07/28-2020/8/05

Small size expression for protein identification.

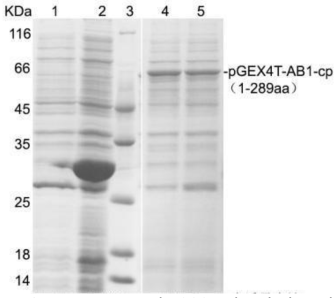

1: pGEX4T-AB1-cp(1-289 aa) uninduced
2: pGEX4T-AB1 empty vector induced
3: Marker
4: pGEX4T-AB1-cp(1-289 aa) induced
5: pGEX4T-AB1-cp(1-289 aa) induced

Result analyses:

Small size expression analysis showed that the target protein has been expressed successfully with the size of about 60 KD.

Identification of target protein after disruption of bacteria and purification

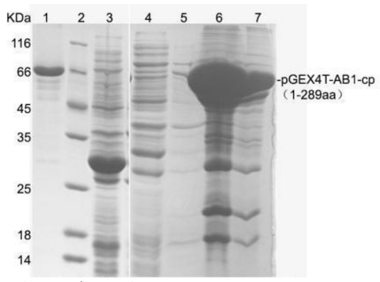

1: 0.4 mg/mL BSA
2: Marker
3: pGEX4T-AB1 empty vector induced
4: Supernatant
5: Supernatant2 (dissolved in 2M urea)
6: 2-time diluted inclusion bodies (dissolved in 8M urea)
7: 10-time diluted inclusion bodies (dissolved in 8M urea)

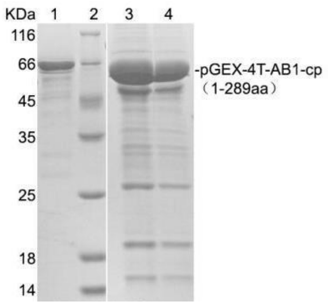

1: 0.4 mg/mL BSA
2: Marker
3: 2-time diluted inclusion bodies (dissolved in 8M urea)
4: 10-time diluted inclusion bodies (dissolved in 8M urea)

Result analyses:

1. pGEX-4T-AB1-CP (1-2889 aa) was expressed in inclusion bodies.
2. The concentration of the inclusion bodies was 15 mg/mL, the purity of the inclusion bodies is sufficient for immunization, and the inclusion bodies was used for immunization.

2.2 免疫流程

Time of Immunization	Cycle of Immunization	Time of Immunization	Dose of Immunization	Immune adjuvant	Animal status
Primary immunization	1 day	2020/8/7	0.3mg	FREUD'S ADJUVANT, COMPLETE	good
Secondary immunization	12 days	2020/8/19	0.15mg	FREUD'S ADJUVANT, INCOMPLETE	good
Third immunization	26 days	2020/9/2	0.15mg	FREUD'S ADJUVANT, INCOMPLETE	good
Fourth immunization	40 days	2020/9/16	0.15mg	FREUD'S ADJUVANT, INCOMPLETE	good
Blood collection	52 days	2020/9/28			Normal 1

2.3 抗血清 ELISA 检测数据

ELISA Coating: pGEX-4T-AB1-CP（1-289aa）

Coating concentration: 2ug/mL, 100ul/well, in CB buffer

Secondary antibody: Peroxidase-conjugated AffiniPure Goat Anti-Rabbit IgG (H+L)
Dilution of secondary antibody: 1:8000

WG-04332D cp	Blank	Negative Control 1:1K	Negative Control 1:64K	Positive 1:1K	Positive 1:4K	Positive 1:8K	Positive 1:16K	Positive 1:32K	Positive 1:64K	Positive 1:128K	Positive 1:256K	Positive 1:512K
E15946	0.0428	0.0384	0.0364	1.0447	1.0059	0.8967	0.7498	0.5664	0.4067	0.2786	0.1691	0.1358
E15947	0.0557	0.0199	0.0264	1.0323	0.9669	0.9026	0.76	0.5768	0.4228	0.2824	0.1649	0.1319

Result Analyses: When coated with 200 ng antigen, ELISA O.D. values of antiserum from both E15946 and E15947 that were immunized for four times were greater than 0.4 when dilution at 1:64000, indicating the antiserum titer is qualified.

2.4 Antiserum purification

1) Detection of protein for affinity purification

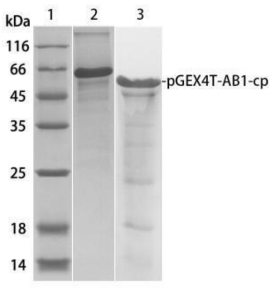

1.Marker
2.0.4mg/mL BSA
3.pGEX4T-AB1-cp diluted in 10 folds

Result Analyses: The concentration of pGEX-4T-AB1-CP protein used for affinity purification was 4 mg/mL, the concentration and purity of which was similar to that after bacteria-breaking disruption and purification, and can be used for antigen-affinity purification.

2) Antiserum purification

After antigen-affinity purification with pGEX-4T-AB1-CP protein, the concentrated antibodies were obtained:

E15946: Concentration 1.92 mg/mL

E15947: Concentration 2.57 mg/mL

2.5 Detection of antigen or endogenous protein by WB

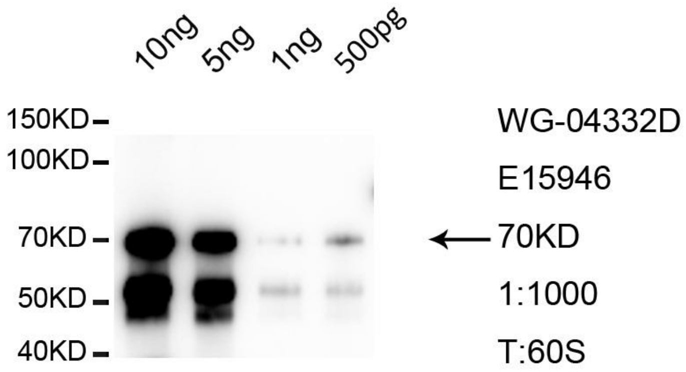

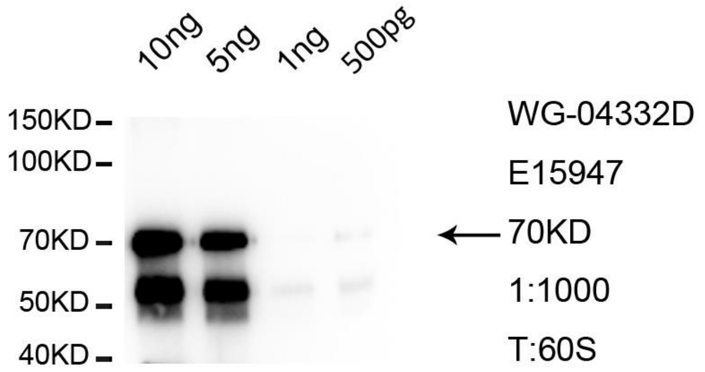

Description: Each lane in the figure is 10 ng, 5 ng, 1 ng, 500 pg antigen, respectively; The dilution of antibodies was 1:1000.

Result Analyses:

1) Antibodies of E15946 and E15947 detects the antigen at a band of about 70 KD;
2) Antibodies of E15946 and E15947 can detect 5 ng antigen at a dilution of 1:1000;
3) Concentrations of E15946 and E15947 antibodies were normal.

3、Notes on the use and preservation of antibodies

WB dilution: 1/500-1/1000

Storage conditions: Store at -20℃ to avoid repeated freeze-thaw

Buffer liquid system: PBS, 50% glycerol, pH7.3
